# Experimental Composite Resin with Myristyltrimethylammonium Bromide (MYTAB) and Alpha-Tricalcium Phosphate (α-TCP): Antibacterial and Remineralizing Effect

**DOI:** 10.3390/jfb14060303

**Published:** 2023-06-01

**Authors:** Juan Carlos Pontons-Melo, Gabriela de Souza Balbinot, Salvatore Sauro, Fabrício Mezzomo Collares

**Affiliations:** 1 Department of Dental Materials, School of Dentistry, Federal University of Rio Grande do Sul. Ramiro Barcelos Street, 2492, Rio Branco, Porto Alegre 90035-003, RS, Brazil; drjcpontons@hotmail.com (J.C.P.-M.); gabriela.balbinot@ufrgs.br (G.d.S.B.); 2 Dental Biomaterials and Minimally Invasive Dentistry, Department of Dentistry, Cardenal Herrera-CEU University, CEU Universities, C/Santiago Ramón y Cajal, s/n., Alfara del Patriarca, 46115 Valencia, Spain

**Keywords:** composite resin, dental caries, streptococcus mutans, antibacterial agents, bioactive materials

## Abstract

The aim of this study was to develop an experimental composite resin with the addition of myristyltrimethylammonium bromide (MYTAB) and α -tricalcium phosphate (α-TCP) as an antibacterial and remineralizing material. Experimental composite resins composed of 75 wt% Bisphenol A-Glycidyl Methacrylate (BisGMA) and 25 wt% Triethylene Glycol Dimethacrylate (TEGDMA) were produced. Some 1 mol% Trimethyl benzoyl-diphenylphosphine oxide (TPO) was used as a photoinitiator, and butylated hydroxytoluene (BTH) was added as a polymerization inhibitor. Silica (1.5 wt%) and barium glass (65 wt%) particles were added as inorganic fillers. For remineralizing and antibacterial effect, α-TCP (10 wt%) and MYTAB (5 wt%) were incorporated into the resin matrix (α-TCP/MYTAB group). A group without the addition of α-TCP/MYTAB was used as a control. Resins were evaluated for their degree of conversion (n = 3) by Fourier Transform Infrared Spectroscopy (FTIR). The flexural strength (n = 5) was assessed based on ISO 4049:2019 requirements. Microhardness was assessed to calculate softening in solvent (n = 3) after ethanol immersion. The mineral deposition (n = 3) was evaluated after immersion in SBF, while cytotoxicity was tested with HaCaT cells (n = 5). Antimicrobial activity (n = 3) was analyzed against *S. mutans.* The degree of conversion was not influenced by the antibacterial and remineralizing compounds, and all groups reached values > 60%. The α-TCP/MYTAB addition promoted increased softening of polymers after immersion in ethanol and reduced their flexural strength and the viability of cells in vitro. A reduction in *S. mutans* viability was observed for the α-TCP/MYTAB group in biofilm formation and planktonic bacteria, with an antibacterial effect > 3log_10_ for the developed materials. Higher intensity of phosphate compounds on the sample’s surface was detected in the α-TCP/MYTAB group. The addition of α-TCP and MYTAB promoted remineralizing and antibacterial effects on the developed resins and may be a strategy for bioactive composites.

## 1. Introduction

Advances in composite resin formulations have been made over the years to address clinical challenges. In current times, this material occupies a prominent place among restorative materials due to its aesthetics, adhesion, and application in conservative techniques [1,2]. Although these materials have a major role in the re-establishment of tooth function and aesthetics, annual failure rates (AFR) range between 0.08% to 4.9% for posterior restorations and 1.4% and 6.3 for anterior restorations. Most failures are associated with secondary caries [3]. Patient-level factors play an important role in these failures, and high-risk patients are more prone to present treatment failure [4]. Promoting a local microenvironment to avoid biofilm accumulation and hard tissue demineralization could contribute to tissue damage control in these cases.

Efforts have been made to incorporate antibacterial and remineralizing agents into composite restorative materials [5,6]. The antibacterial effect of composites would be principally significant to the inhibition of biofilm accumulation on the surface of the tooth and restorative materials [5]. The addition of antimicrobial compounds has been used as a strategy to control biofilm impact in areas close to restorations [6]. Quaternary ammonium compounds (QACs) are among the antibacterial agents applied in this case, being studied to produce composite resin [7,8], orthodontic adhesives [9], adhesive resins [10], and dental sealants [11]. The myristyltrimethylammonium bromide (MYTAB) is a QAC with an optimal 14-chain carbon length and a cation head that promotes the antibacterial effect found in this compound [12,13]. This structure gives a tensioactive characteristic to MYTAB and the alternation of cell metabolism, cytokinesis, and endocytosis pathways in bacterial cells with a low risk for environmental antibacterial resistance due to its non-specific cell wall disruption with a broad spectrum of action [14,15]. When applied to dental adhesives, this compound promoted inhibition of biofilm viability and growth [16] with a reduction in bacteria viability when applied in irrigating solutions [17].

The tissue damage in caries-affected teeth is related to an imbalance in the demineralization–remineralization process, mediated by the adhered biofilm [18,19]. In addition to the reduction in biofilm formation, establishing a healthy demineralization–remineralization process could contribute to a healthier tooth–resin interface. Ion-releasing inorganic particles are mainly studied for this aim [20,21,22]. Variations in bioactive glasses [23,24] and calcium phosphate [25,26] compositions are tested for improving the available Ca^2+^ and PO_4_^3−^ ions that are available for ion exchange in the microenvironment [20]. Among various CaP compounds, α-tricalcium phosphate (α-TCP) is a reactive compound with high biodegradation rates and interacts with tissues to stimulate mineral deposition via controlled solubilization of calcium and phosphate [27,28]. The degree of α-TCP controlled solubility was shown to control the impact of calcium phosphate addition into methacrylate-based composites, with reduced long-term impact on the properties of the materials [27,28,29]. The combination of the antibacterial effect of MYTAB with the remineralizing properties of α-TCP could lead to the ability to protect and remineralize tooth structure while inhibiting biofilm formation, contributing to a controlled microenvironment in areas that may present a high risk for secondary caries development. Thus, the aim of this study was to develop an experimental composite resin with the addition of myristyltrimethylammonium bromide (MYTAB) and α-tricalcium phosphate (α-TCP) as an antibacterial and remineralizing material.

## 2. Materials and Methods

**Figure 1:**
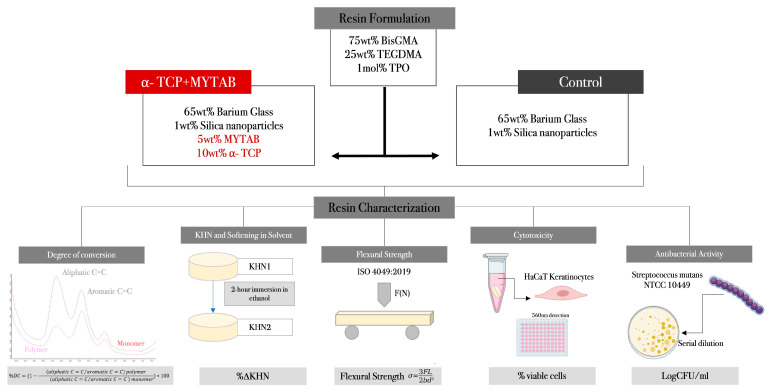
Schematics of study design, resin composites formulation in each experimental group, and the methods applied for characterization.

The study design, formulation of experimental groups, and representation of resin characterization are shown in Figure 1.

### 2.1. Study Design and Formulation of Experimental Resin

The experimental composite resins were formulated, as described previously, with Bisphenol A-Glycidyl Methacrylate (BisGMA, 75 wt%) and Triethylene Glycol Dimethacrylate (TEGDMA, 25 wt%). As a photoinitiator system, mono-alkyl phosphine oxide (TPO) was added at 1 wt%, while butylhydroxytoluene (BTH) was added at 0.01 wt% as an inhibitor [9,30]. As inorganic fillers, barium silicate glass (65 wt%) and nanosized silica (1 wt%) were added. For the α-TCP/MYTAB (α-TCP/MYTAB) composites, the alpha-tricalcium phosphate (α-TCP) [31] was added at 10 wt%, while the myristyltrimethylammonium bromide (MYTAB) with a purity of >99% (crystalline powder) was added at 5 wt%. A composite resin without MYTAB and α-TCP addition was used as a control. All components were weighed in an analytical balance (AUW220D; Shimadzu, Kyoto, Japan). After being hand-mixed for 5 min, they were sonicated with an ultra-sonic tip (Sonic Vibra Cell. Sonic & Materials Inc., Newtown, CT, USA. Model: VCX 130) with a 10% amplitude. Then, a 21 A light-curing unit (VALO Cordless, Ultradent Products, Salt Lake City, UT, USA) was used for the activation of specimens at 1000 mW/cm^2^. All reagents were acquired from Sigma-Aldrich (Sigma-Aldrich Chemical Company, St. Louis, MO, USA).

### 2.2. Degree of Conversion (DC)

Fourier transform infrared spectroscopy (FTIR, Vertex 70, Bruker Optics, Ettinger, Germany) was used to evaluate the degree of conversion in the experimental resin composites. The unpolymerized dental resins (n = 3) were placed on an attenuated total reflectance (ATR) device inside a polyvinylsiloxane matrix measuring 1 mm in thickness and 4 mm in diameter [32]. The specimens were covered with polyester matrix strips to obtain the FTIR spectra of unreacted specimens. The specimens were then photoactivated using a light-curing unit for 40 s, and other spectra were obtained. The spectra were obtained with a 4 cm^−1^ resolution in the spectral range between 400 and 4000 cm^−1^ with a mirror speed of 2.8 mm/s using Opus software (Opus 6.5, Bruker Optics, Ettlingen, Germany). The DC was calculated considering the intensity of the carbon–carbon double bond (peak at 1640 cm^−1^) and using the aromatic carbon–carbon double bond (peak at 1610 cm^−1^) as an internal standard [33].

### 2.3. Knoop Microhardness and Softening in Solvent

Specimens (n = 3) were prepared (1.0 mm thickness × 4.0 mm diameter) with photoactivation for 20 s on each side and polished with silicon carbide sandpapers, followed by felt discs with an alumina suspension 28 before the analysis. Knoop microhardness was performed on a microhardness tester (HMV 2; Shimadzu, Tokyo, Japan) with 10 sg for 5 s. Five indentations were performed per sample. After KHN1, the specimens were stored in a 70% ethanol solution for 2 h. The specimens were then submitted to a final KHN test (KHN2) using the same conditions. The difference between KHN1 and KHN2 was used to calculate de ∆KHN% for softening solvent analysis.

### 2.4. Flexural Strength

The flexural strength test was conducted according to ISO 4049 [34]. Six rectangular mini-flexural specimens (n = 6) per group were prepared in a metallic mold that was 12.0 mm long, 2.0 mm wide, and 2.0 mm thick [35]. For the preparation of the specimens, the dental resin was dispensed into the mold and covered with polyester matrix strips. Polymerization was performed in two windows for 20 s on each side of the specimen. The prepared specimens were stored in distilled water at 37 °C for 24 h. The specimens were submitted under tensile strength in a universal testing machine (EZ-SX Series, Shimadzu, Kyoto, Japan) at 1 mm/min until fracture. Flexural strength was calculated from the following equation:

$$\;\sigma =\frac{3LF}{2BH^{2}}$$

where *σ* is the flexural strength (MPa), *F* is the maximum load (N), *L* is the distance (mm) between the holders (up to 0.01 mm), *B* is the width (mm) at the center of the specimen, and *H* is the height (mm) of the specimen, measured with a digital caliper just before the test.

### 2.5. Cytotoxicity

Specimens (n = 5) were prepared with the aid of a silicon mold (1 mm thickness × 4 mm diameter) and photoactivated for 20 s on each side.31 Human keratinocytes (HaCaT, CLS Cell Lines Service GmbH, Eppelheim, Germany) were used in this test. Then, 32 The HaCaT cells were cultivated at 5 × 103 cell density in supplemented Dulbecco’s modified Eagle’s medium (DMEM) in 96-well plates for 24 h at 37 °C and 5% CO_2_. Extracts were prepared by immersing samples in culture media for 24 h and then used to treat HaCaT cells for 72 h at 37 °C. The culture was performed in triplicate. After treatment, the cell monolayer was fixed and stained with sulforhodamine B at 0.4% (SRB) to assess the absorbance of each well at 560 nm. The absorbance was used to calculate the percentage of viable cells obtained via the normalization of results considering the absorbance of cells that were not treated with the extracts of the material.

### 2.6. Antibacterial Activity

The antibacterial activity was tested with a Streptococcus mutans (NCTC 10449) culture. *S. mutans* were prepared according to a previous study, 28 with an inoculum concentration of 7.8 × 107 CFU/mL. In order to evaluate the antibacterial activity against biofilm formation, the polymerized specimens (1 mm thickness × 4 mm diameter) were fixed on Teflon, which was fixed on the cover of a 48-well plate. From the initial inoculum, 100 μL was added in each well of a 48-well plate with 900 μL of brain–heart infusion (BHI) broth with 1 wt% of sucrose. The sterile set of cover and specimens was joined with this 48-well plate and kept for 24 h under 37 °C for biofilm formation. After this period, each specimen was detached from the cover and vortexed for 1 min in 1 mL of sterile saline solution. The solution was serial diluted up to 10^−6^ mL and plated on Petri dishes containing BHI agar to count the colonies and to calculate the colony-forming units per milliliter (CFU/mL). To assess the antibacterial activity against planktonic bacteria, the BHI broth that was in contact with the polymerized specimens along the 24 h mentioned above was used. From each well of the 48-well plate, 100 μL was collected after the 24 h of bacteria–specimen contact, diluted until 10^−6^ mL, and plated on BHI agar Petri dishes. Negative control was used on the planktonic analysis using the same conditions but without contact with materials.

### 2.7. Mineral Deposition

Mineral deposition analysis was performed using Raman spectroscopy (Senterra, Bruker Optics, Ettingen, Germany), equipped with a 100-mW diode laser adjusted to a 785-nm wavelength with a spectral resolution of ≈3.5 cm in three co-additions for five seconds. Specimens (4 mm diameter × 1 mm height) were scanned in a 1 mm 2 area where 100 equidistant points were analyzed. After an initial analysis, samples were immersed in simulated body fluid (SBF) at 37 °C. Specimens were then analyzed again, using the same conditions at 7, 14, 21, and 28 days. The obtained Raman spectra were used to detect the phosphate peak (960 cm^−1^). The peak was integrated, and its height was measured for each analyzed point. The measurements were used to plot color maps where differences in the phosphate intensity over the sample are represented by different colors on all the analyzed areas.

### 2.8. Statistical Analysis

A descriptive analysis was conducted to evaluate the mineral deposition results. Data were analyzed by the Shapiro–Wilk test for normality. Student’s *t*-Test for independent samples was used to compare differences between groups in the degree of conversion, flexural strength, cytotoxicity, KHN1, and ∆KHN%. Differences between KHN1 and KHN2 in each group were assessed by the Students *t*-Test for dependent samples. Descriptive analysis was performed for mineral deposition results. All tests were performed at a 5% significance level.

## 3. Results

**Figure 2:**
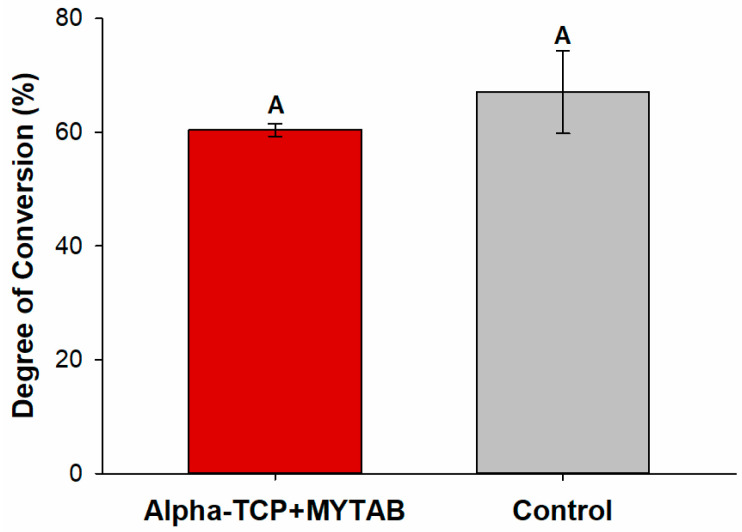
Degree of conversion (%) for analyzed groups. Different capital letters indicate a statistically significant difference between different groups.

Figure 2 shows that the incorporation of α-TCP+MYTAB into the BiSGMA: TEGDMA resin composite did not affect the DC. The values ranged from 67.08 (±7.25) for α-TCP+MYTAB to 60.41 (±1.10) for the Control group.

The KHN1 showed no statistically significant difference between the analyzed groups (Table 1). All groups decreased the microhardness after storage for 2 h in ethanol (*p* < 0.05) with a%ΔKHN ranging from 25.49 (±2.14) for α-TCP/MYTAB to 12.17 (±3.11)% for the Control group, indicating that the presence of antibacterial particles led to an increased softening in solvent outcome (*p* < 0.001).

**Figure 3:**
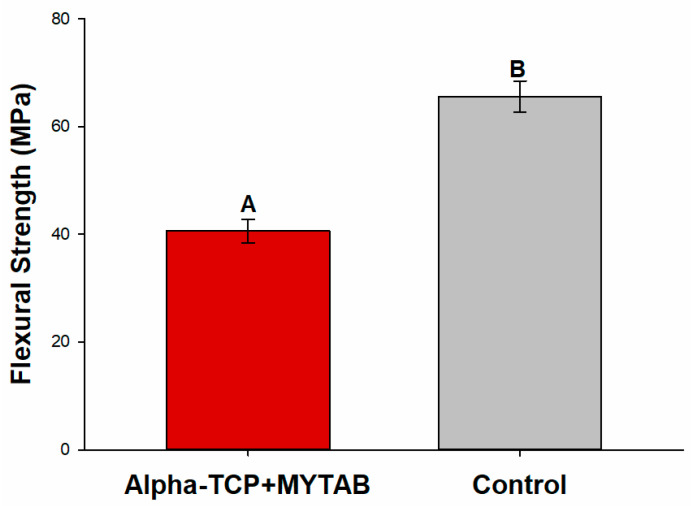
Flexural strength results obtained from ISO 4049:2019 standardized analysis. Different capital letters indicate a statistically significant difference between different groups.

A higher flexural strength was observed for the Control group (Figure 3; *p* > 0.05). The average values of α-TCP/MYTAB were 40.61 (±2.15) while the control group presented 65.60 (±2.90) MPa, both values that are lower than the required by ISO 4049:2019.

**Figure 4:**
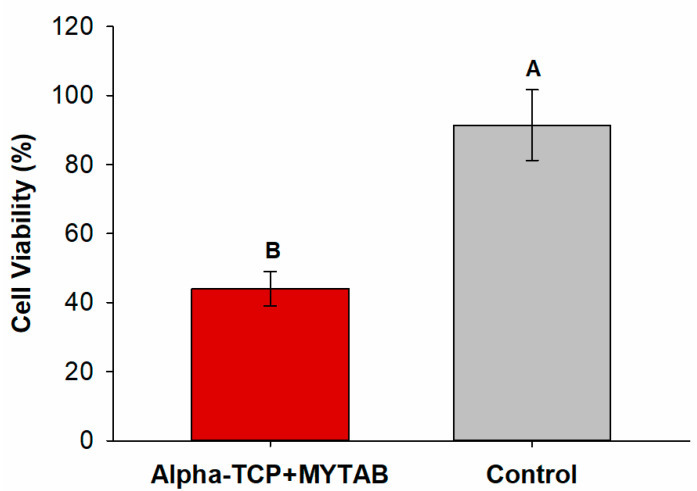
Cytotoxic analysis of α-TCP/MYTAB and the Control group. Different capital letters indicate a statistically significant difference between different groups.

Keratinocytes (HaCaT) viability after SRB assay is observed in Figure 4. After 72 h of contact between the eluates and the cells, the viability percentage ranged from 91.44 (±10.22)% for Control to 43.33 (±4.98)% for α-TCP/MYTAB (*p* < 0.001), with a statistical difference between groups.

For the antibacterial assay, the Control group presented increased CFU/mL on biofilm and planktonic analysis when compared to α-TCP/MYTAB group. As shown in Table 2, no colony-forming units were detected in biofilm analysis on the surface of α-TCP/MYTAB-loaded resin composites, while a > 3log reduction was observed for planktonic bacteria.

**Figure 5:**
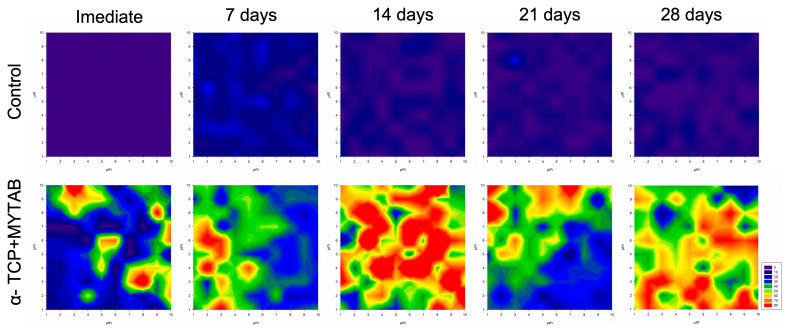
Mineral deposition: the phosphate peak (960 cm^−1^) quantification is shown in the color map from Raman intensity with the high content of mineral represented by red/orange color, whereas blue/purple represents the absence of phosphate on the specimens.

The mineral deposition was increased immediately and after the immersion of specimens in SBF for the α-TCP/MYTAB, as shown in Figure 5. The phosphate peak (960 cm^−1^) was assessed immediately and after 7, 14, 21, and 28 days of immersion of specimens in SBF, and its quantification is shown in the color maps. At initial analysis, the control group presented low phosphate intensity, which is assigned for purple/blue regions in the mapping. The presence of phosphates in the α-TCP/MYTAB is observed in the immediate analysis, where green, orange, and red areas show the increase in phosphate intensity in certain regions of the screened area. While low phosphate intensities were maintained over the analyzed times, up to 28 days (blue maps), increases in the area covered by high-intensity phosphate peaks are observed at 14 days in the α-TCP/MYTAB group.

## 4. Discussion

The antibacterial and remineralizing activity of resin composites was obtained in the present study with the addition of MYTAB and α-TCP. The filler and molecule load resulted in the ability of these materials to reduce the biofilm formation on the specimens while stimulating the formation of phosphate groups on their surface. These characteristics could lead to a more stable microenvironment during the demineralization–remineralization process.

MYTAB and α-TCP incorporation into a dental resin formulation resulted in no reduction of the degree of conversion, and all groups presented at least 60% DC (*p* > 0.05; Figure 2). The DC of a resin composite is indispensable to achieving optimal physical and mechanical properties [36,37]. Strength, modulus, hardness, solubility, and reduction of possible cytotoxic effects have been shown to be directly related to the degree of monomer conversion [38,39,40]. Modifications in the refractive index among the compounds added, and the resins, the increase of resins viscosity, and monomers mobility may affect the DC and the polymerization kinetics and except for the addition of α-TCP, as an opaque crystalline compound, has not affected the light transmittance over the irradiated samples when compared to the control group with barium silicate glass as filler. Though appropriate DC helps improve the mechanical properties of polymers, it does not elucidate the network quality. The presence of antibacterial monomers, such as the MYTAB, may also modify the formation of polymeric structures [9,10,41]. Their effect on the resistance of polymers to softening, as shown in Table 1, was influenced by the addition of α-TCP/MYTAB. After comparable KHN1 values, both groups presented a reduction in KHN values after solvent immersion, with higher% ΔKHN for α-TCP/MYTAB group. As a non-copolymerizable quaternary ammonium compound, MYTAB is not expected to increase in cross-linking density in the polymerized composites, while it may be related to changes in the methacrylate chain formation and in the secondary interaction between them, which could reduce the polymer’s resistance to softening [42,43].

The material resistance was also affected by α-TCP/MYTAB addition (Figure 3). Both the α-TCP and the MYTAB may impact the tension distribution over the resins during mechanical loading. The interaction of α-TCP with the methacrylate matrix is poor as bonding agents are not effective due to the calcium phosphate composition [44]. The presence of these particles in the composites may lead to the tension concentration in the organic–inorganic interface in the composite, leading to their failure, an effect that is known for several remineralizing fillers and was expected with the incorporation of these fillers. The ratio between α-TCP and barium glass fillers in the α-TCP/MYTAB group was adjusted to balance the mechanical and biological properties, as observed in the deposition of minerals in Figure 5. Obtaining bioactive composites with adequate mechanical behavior is a challenge, and strategies to promote a better interaction between matrix and fillers have been proposed [25,32]. Flexural strength is usually performed based on ISO 4049 requirements [34], and none of the studied groups reached the standard values of 80 MPa for the produced class of materials. It is important to highlight that, in this study, mini-flexural specimens were used, as the ISO bar-shaped specimens may be technically difficult to prepare without flaws. The longer specimens require various overlapping light irradiations, and commercial curing lights are typically less than 25 mm, leading to a higher probability of low irradiation of some regions during the photoactivation for specimen production. Although it may not be possible to directly compare the values to the ISO requirements, the reduction in mechanical properties for the bioactive materials was observed.

The MYTAB addition was probably responsible for the findings of cytotoxicity (Figure 4). A reduction in cell viability results was observed for the α-TCP/MYTAB. The cytotoxicity of these materials may be related to the release of unreacted monomers and other resin products. In this case, as MYTAB does not interact with the methacrylate matrix, their release may be facilitated, leading to the protein viability reduction of the studied cells (*p* > 0.05; Figure 4). Most quaternary ammonium compounds do not present a cytotoxic effect [45], even when analyzed in dental composites [7,8,9,15]. The MYTAB addition to dental adhesives, however, promoted a reduction in cell viability of dental pulp cells [16], and while the antibacterial effect of MYTAB is known, it is not clear how this compound affects eukaryotic cell viability. Considering the previous findings on MYTAB cytotoxicity, control of MYTAB concentration in the developed composites could be a strategy to reduce the effect of materials in the analyzed cells. The HaCaT cell line was used in this study as a model to understand the potential contact with resin products related to composites and the surrounding oral mucosa [46]. Although a reduction in cell viability was found for the resins with MYTAB, it is important to highlight that direct–indirect cytotoxic assays overestimate the effect of these materials in contact with two-dimensional cell cultures and when in resin composites [47]. In addition, as resins are not directly placed over soft tissues, a low release is expected, limiting the impact of the released products on the viability of surrounding cells. The establishment of an adequate polymer matrix may contribute to limiting the dose of these materials in contact with cells, and their release to the oral cavity may control a possible dose–response effect. Controlling the concentration of MYTAB may thus be a strategy to overcome this limitation and adjust the biological properties of the designed material.

The ability of MYTAB to directly disrupt the microbial cell membrane leading to cell death with a minor effect on bacterial resistance due to a non-specific and broad spectrum, is desired for the formulation of antibacterial compounds [13]. In this study, the polymerized resins were in contact with an enriched broth containing *S. mutans*, and α-TCP/MYTAB reduced the biofilm formation on the resin composite surface while reducing the viability of planktonic bacteria. As an initial screening of in vitro antibacterial activity, these results show effective control of bacteria promoted by the developed resins. These outcomes are related to the positively charged nitrogen (N+) head and the long alkyl chain with 14 carbons. It is known that longer alkyl chains are related to better hydrophobicity in these compounds, and thus, the longer the alkyl chain, the higher the ability of this compound to degrade bacterial membranes [15]. The control on both the deposition of S.mutans on resin samples and the control on bacteria in the surrounding of restorations could be used to avoid bacteria colonization over directly-placed restorations in high-risk areas where non-invasive measurements are not effective and for patients that are not apple to comply with hygiene and biofilm control measurements.

High-risk areas may be more prone to lose mineral content in regions nearby restorations and thus the mineral deposition stimulated by bioactive dental materials might be an alternative to reduce tissue loss and failure due to secondary caries lesions [48,49]. The incorporation of α-TCP in a composite resin could be an alternative to add the remineralization ability of the resins, along with the antibacterial effect aiming to improve composite-teeth interface resistance. Calcium phosphates have been extensively studied in composite resins for their release of high levels of calcium and phosphate ions, being able to remineralize dentin and enamel lesions [50]. The mineral deposition was increased after the immersion of α-TCP/MYTAB of specimens in SBF (Figure 5). The PO43-vibrations are observed in the non-immersed specimens as part of the α-TCP composition, and after selected time points, the increased intensity is observed on the red/orange areas, assigning higher intensities on the 960 cm^−1^ peak on the Raman mapping (Figure 5). These results are corroborated by findings from other studies that show α-TCP as a candidate for remineralization therapies [28,51]. The α-TCP solubility may be responsible for the maintenance of the effect for over 28 days, which is desired as a long-term effect is necessary for restorative treatments. These results may be confirmed by pre-clinical and in vivo data to further assess the ability of these materials to control the demineralization process.

The balance between the material’s physicochemical properties and its ability to control the negative impacts of dental biofilm in the tooth-restoration interface is a challenge for newly developed materials. In this study, we show that the development of composite resins with potential antibacterial and remineralizing effects is possible, but an impact on the mechanical behavior and biocompatibility was observed. The screening of antibacterial and remineralizing agents for dental resins using different concentrations can contribute to understanding the dose–response effect and provide for reducing the drawbacks of the material’s properties, contributing to the establishment of suitable properties towards the clinical application of dental composites with therapeutic activity. This may help the development of tailored strategies for high-risk individuals that may benefit from additional control of biofilm formation and tooth remineralization via restorative materials.

## 5. Conclusions

The addition of α-TCP and MYTAB promoted remineralizing and antibacterial effects on the developed resins and may be a strategy for bioactive composites, with adjustments on the biological and mechanical response for an adequate balance between properties for clinical application.

## Figures and Tables

**Table 1:** Softening solvent results. The%ΔKHN was calculated based on the difference between microhardness before and after immersion in ethanol.

	KHN1	KHN2	%ΔKHN
α-TCP/MYTAB	80.49 (±7.11) Aa	59.92 (±5.10) b	25.49 (±2.14) B
Control	73.28 (±4.98) Aa	64.33 (±4.20) b	12.17 (±3.11) A

Different capital letters indicate a statistically significant difference between different groups. Different small letters indicate a statistically significant difference between KHN1 and KHN2.

**Table 2:** Mean and standard deviation values of log-transformed colony-forming units/mL (CFU/mL) for bacteria in biofilm and planktonic bacteria after contact during 24 h with the experimental dental resins.

	Antibacterial Activity (log CFU/ML)	
	Biofilm	Planktonic
α-TCP/MYTAB	0.00 (±0.00) B	1.28(±2.22) A
Control	6.90 (±0.44) A	5.87(±0.87) B
Negative Control	-	6.85 (±0.90) B

Different capital letters indicate a statistically significant difference between different groups.

## Data Availability

Data available on request.
